# Additional Diffusion-Weighted Imaging with Background Body Signal Suppression (DWIBS) Improves Pre-Therapeutical Detection of Early-Stage (pT1a) Glottic Cancer: A Feasibility and Interobserver Reliability Study

**DOI:** 10.3390/diagnostics12123200

**Published:** 2022-12-16

**Authors:** Stephan Schleder, Matthias May, Werner Habicher, Johannes Dinkel, Andreas G. Schreyer, Antoniu-Oreste Gostian, Andreas Schicho

**Affiliations:** 1 Department of Diagnostic and Interventional Radiology, Merciful Brothers Hospital St. Elisabeth, 94315 Straubing, Germany; 2 Department of Urology, Merciful Brothers Hospital St. Elisabeth, 94315 Straubing, Germany; 3 Department of Otorhinolaryngology, Merciful Brothers Hospital St. Elisabeth, 94315 Straubing, Germany; 4 Department of Radiology, University Medical Center Regensburg, 93055 Regensburg, Germany; 5 Department of Diagnostic and Interventional Radiology, University Hospital Brandenburg, Brandenburg Medical School Theodor Fontane, 14770 Brandenburg, Germany; 6 Department of Otolaryngology, Head & Neck Surgery, Comprehensive Cancer Center Erlangen, University Hospital Erlangen, 59991 Erlangen, Germany

**Keywords:** MRI, DWIBS, glottic cancer, staging

## Abstract

(1) Background: Early-stage glottic cancer is easily missed on magnetic resonance imaging (MRI). Diffusion-weighted imaging (DWI) may improve diagnostic accuracy. Therefore, our aim was to assess the value of adding diffusion-weighted imaging with background body signal suppression (DWIBS) to pre-therapeutic MRI staging. (2) Methods: Two radiologists with 8 and 13 years of experience, blinded to each other’s findings, initially interpreted only standard MRI, later DWIBS alone, and afterward, standard MRI + DWIBS in 41 patients with histopathologically proven pT1a laryngeal cancer of the glottis. (3) Results: Detectability rates with standard MRI, DWIBS only, and standard MRI + DWIBS were 68–71%, 63–66%, and 73–76%, respectively. Moreover, interobserver reliability was calculated as good (κ = 0.712), very good (κ = 0.84), and good (κ = 0.69) for standard MRI, DWIBS only, and standard MRI + DWIBS, respectively. (4) Conclusions: Standard MRI, DWIBS alone, and standard MRI + DWIBS showed an encouraging detection rate, as well as distinct interobserver reliability in the diagnosis of early-stage laryngeal cancer when compared to the definitive histopathologic report.

## 1. Introduction

Laryngeal cancer is attributed to the second-highest rate of new cases of head and neck carcinomas, and the global incidence is rising, especially in Europe and the United States of America [1,2]. Most laryngeal cancers are associated with tobacco and alcohol consumption, and more than 90% of all laryngeal cancers are squamous cell carcinomas (SCC), followed by adenocarcinomas. In contrast to oropharyngeal cancers, infection with the human papilloma virus does not play any significant role in its origin [3,4,5]. Early detection of glottic carcinoma is of utmost importance, allowing 5-year survival rates of more than 90%. The diagnostic work-up of patients with suspected cancer of the larynx has long remained unchanged. It comprises an endoscopic biopsy and a multislice computed tomography (MSCT) of the neck to delineate the tumor spread and detect locoregional nodal metastases [5]. However, magnetic resonance imaging (MRI) is now becoming the method of choice due to its superior soft-tissue contrast, not only for precise localization of the primary tumor but also for the recognition of tumor invasion of adjacent areas of the larynx [5]. Advances in MRI compared to CT are not limited to the superior soft-tissue contrast enabling the detection of even subtle soft-tissue abnormalities. MRI enables increased discrimination between the tumor and peritumoral inflammatory changes, better assessment of laryngeal cartilage abnormalities, improved tumor delineation in preparation for highly focused radiotherapy and transoral laser microsurgery, and higher diagnostic performance for the detection and precise depiction of posttreatment residual or recurrent disease, especially when diffusion-weighted imaging (DWI) is included [5,6,7,8,9,10,11]. 

Nevertheless, assessment of early-stage laryngeal cancer of the glottis by MRI remains a challenge [1,5,12] as some drawbacks of MRI persist compared to CT, including a longer acquisition time and motion artifacts requiring improved patient cooperation, a lower spatial resolution, and technical challenges related to air-tissue interfaces affecting the image quality of DWI sequences [5,13].

Addressing the limitations of traditional DWI imaging methods, Takahara et al. [14] developed a novel imaging method, “diffusion-weighted imaging with background signal suppression (DWIBS)”, to obtain thin-layer DWI [13,14]. DWIBS can provide multi-level stimulation and average signal times in a slightly longer time and can effectively inhibit fat by using short T1 inversion recovery (STIR)-echo planar imaging (EPI) sequence, all of which improve the quality of 3D reconstruction images of (whole-body) imaging [13,14]. 

DWIBS demonstrates good background signal suppression, which emphasizes the lesions and lymph nodes in a stereoscopic and intuitive way. Through the black-and-white reversal technology, DWIBS obtains PET-like images. DWIBS has been widely applied in the clinical diagnosis of brain, nerve, and abdominopelvic diseases, as well as in the staging routine of various cancer entities [15,16,17,18,19,20,21]. 

For early-stage laryngeal cancer affecting the vocal cord only, there is, to date, no study evaluating the diagnostic accuracy and interobserver reliability of DIWBS. Therefore, our aim was to assess the value of adding DWIBS to pre-therapeutic MRI staging.

## 2. Materials and Methods

### 2.1. Patients

**Figure 1:**
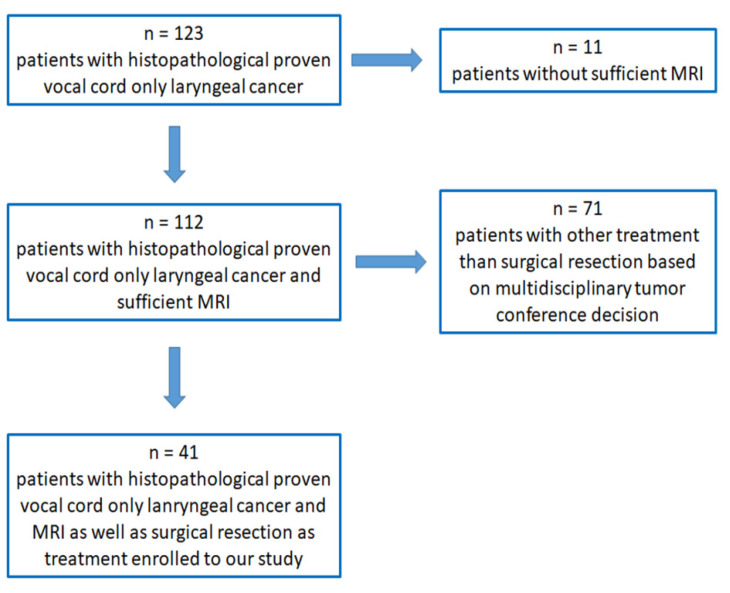
Scheme of enrolled patients with histopathologically proven vocal cord-only laryngeal early-stage cancer (pT1a) for further analysis.

The study was approved by the institutional ethics committee of the Medical Faculty of the University of Regensburg (No. 19-1492-104), and written informed consent was obtained from all patients. We identified 123 consecutive patients with newly diagnosed laryngeal cancer fulfilling both in- and exclusion criteria; after a thorough review of every single case (Figure 1), 41 patients were enrolled in our statistical analysis between 1 April 2018 and 31 August 2022 (Figure 1).

Patient inclusion criteria were: (1) Histopathologically proven vocal cord-only laryngeal cancer (pT1a); (2) preoperative staging by MRI including DWIBS, from which clear images without apparent artifacts were obtained; and (3) decision of the multidisciplinary tumor conference for definitive surgical therapy with or without adjuvant chemoradiotherapy 

Patient exclusion criteria were: (1) history of other malignancies; (2) history of neoadjuvant chemoradiotherapy or other treatment for head and neck cancer; and (3) contraindications for MRI including DWIBS (such as severe claustrophobia, ferromagnetic foreign material).

After the initial diagnostic work-up, including histopathology, all patients and cases were discussed at a specialized head and neck multidisciplinary tumor conference before initiation of treatment in a certified head and neck cancer center.

### 2.2. MRI Examination

MRI was performed using a 1.5 Tesla scanner (Ingenia, Philips Medical Systems DMC GmbH, Hamburg, Germany). Studies were performed with a HEAD-NECK COIL system (Philips Medical Systems DMC GmbH, Hamburg, Germany). The patients were placed supine and positioned head-first on the table platform. For acquiring the post-contrast examinations, gadoteric acid (Dotarem, Guerbet Deutschland GmbH, Sulzbach/Taunus, Germany) was injected intravenously, adapted to patient weight at a ratio of 0.2 mL/kg, at a varying dose of 15–20 mL.

Examinations consisted of the following:

(1) Transversal T1-weighted images of the neck without fat saturation (turbospin echo; repetition time (TR) 590, echo time (TE) 10 ms, flip angle 90°) with a slice thickness of 4 mm, section gap of 1 mm, a field of view (FOV) of 260 × 250 × 190 mm, matrix of 430 × 300 × 45 mm, and a scan time of about 2 min 50 s;
(2) Transversal T2-weighted scans of the neck without fat saturation (turbospin echo; TR 9200 ms, TE 100 ms, flip angle 90°) with a slice thickness of 4 mm, section gap of 0 mm, FOV of 260 × 250 × 190 mm, matrix of 320 × 240 × 45 mm, and scan time of about 2 min 55 s;
(3) Diffusion-weighted images with background saturation (DWIBS) in the axial plane with the following parameters: TR 6030 ms, TE 64 ms, flip angle 90°, FOV 260 mm × 250 × 190 mm, matrix 90 × 80 × 50 mm, 4 mm slice thickness, no section gap, b value 0 and 1000 s/mm^2^ and a scan time of approximately 2 min 15 s;
(4) A 3D-Dixon-T1-weighted fat-saturated post-contrast examination of the neck (TR 6 ms, TE 2 ms, flip angle 15°) with a FOV of 260 × 250 × 220 mm, matrix of 260 × 250 × 220 mm and scan time of about 3 min with isotropic voxels of 1 mm and reformatted transversally, sagittally, and coronally with a slice thickness of 3 mm, section gap of 0 mm.

### 2.3. Image Analysis

All acquired images were stored at PACS (Agfa Healthcare Deutschland GmbH, München, Germany) with dedicated diagnostic monitors (MDNC-2221, Barco Deutschland GmbH, Karlsruhe, Germany) prior to surgery and before the histopathologic results were known. Two radiology residents with 8 and 13 years of experience in head and neck radiology, respectively, who were blinded to each other´s findings, interpreted the MR images independently, initially regarding only (1) transversal T1-weighted images, transversal T2-weighted scans, and 3D-Dixon-T1-weighted fat-saturated post-contrast (“standard MRI”); then, separately, (2) diffusion-weighted images with background saturation (“DWIBS”) alone prior to (3) the combination of all images (“standard MRI + DWIBS”). Both readers had to assess whether a tumor lesion was visible (“yes”) or not (“no”).

### 2.4. Statistical Analysis

The statistical analysis was performed using SPSS (version 28, Statistical Package for Social Science, IBM Corp., Armonk, NY, USA). Cohen´s Kappa was used for testing the interobserver reliability of both readers. The level of significance was set at *p* < 0.05. The agreement was interpreted as being poor (κ = 0–0.2), fair (κ = 0.21–0.40), moderate (κ = 0.41–0.60), good (κ = 0.61–0.80), and very good (κ = 0.81–1) [22].

## 3. Results

### 3.1. Patient Characteristics

Of the 41 patients included, 34 were male (83%), and 7 were female (17%), with a median age of 68 years ranging from 37 to 93. Thirty-eight of the forty-one patients (93%) enrolled had the histopathology of squamous cell carcinoma, whereas three patients (7%) were diagnosed with an adenocarcinoma of the vocal cord. A total of 37 patients (90%) had a history of smoking, and a total of 35 patients (85%) reported regular consumption of alcohol. All patients were diagnosed at stage pT1a. Twenty-three carcinomas were detected on the right side (56%) and seventeen on the left (44%).

### 3.2. Findings of Standard MRI 

**Figure 2:**
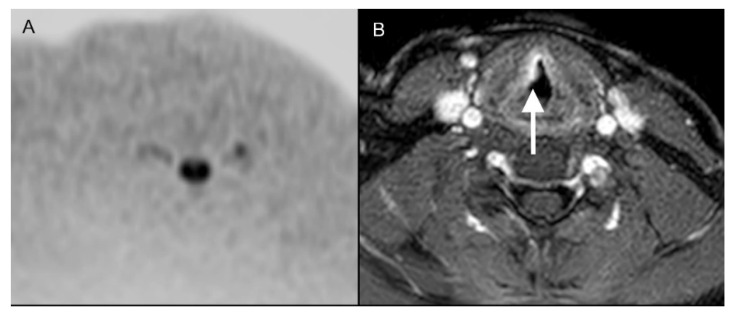
(**A**) A 37-year-old patient with a pT1a cN0 cM0 squamous cell carcinoma of the right vocal cord, which was not detectable in DWIBS (**A**) but was correctly diagnosed in contrast-enhanced transversal T1-weighted scans with fat saturation ((**B**); white arrow) by both readers.

When assessing standard MRI sequences, Reader 1 correctly identified 28 of 41 vocal cord cancers (68%) and missed the diagnosis in 13 cases (32%). In two patients (5%), standard MRI revealed the diagnosis in which Reader 1 did not make the diagnosis with DWIBS alone (Figure 2). Reader 2 correctly detected 29 cases (71%) and did not recognize 12 vocal cord cancers (29%) using standard MRI alone. In four patients (10%), standard MRI revealed the diagnosis in which Reader 2 did not make the diagnosis with DWIBS alone (Figure 2). The interobserver reliability was good (κ = 0.712; *p* < 0.05 (*p* = 4.88 × 10^−^^6^)).

### 3.3. Findings of DWIBS

**Figure 3:**
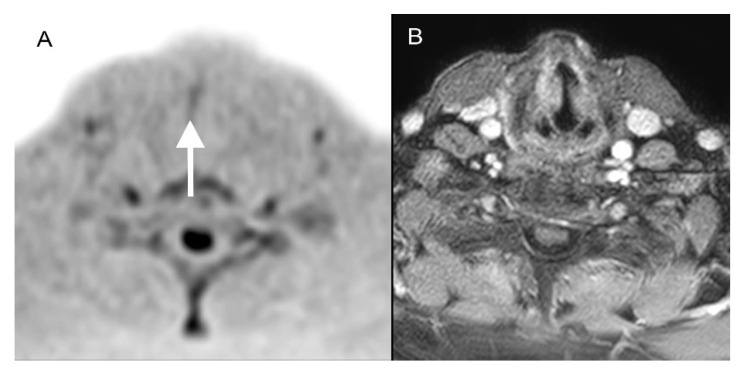
An 80-year-old patient with a pT1a cN0 cM0 squamous cell carcinoma of the vocal cord on the right side, which was diagnosed correctly in DWIBS ((**A**); white arrow) by both readers but not in contrast-enhanced transversal T1-weighted scans with fat saturation (**B**).

Reader 1 found 27 of 41 vocal cord cancers (66%) and missed the diagnosis in 14 cases (34%) when assessing only the DWIBS. In one patient (2.5%), a standard MRI revealed no diagnosis, but Reader 1 did make the diagnosis with DWIBS alone (Figure 3). Reader 2 correctly detected 26 cases (63%) and did not recognize 15 vocal cord cancers (37%) using the standard MRI alone. As with Reader 1, in one patient (2.5%), standard MRI did not show the diagnosis that Reader 2 did make with DWIBS alone (Figure 3). The interobserver reliability was calculated as very good agreement (κ = 0.84; *p* < 0.05 (*p* = 7.18 × 10^−^^8^)).

### 3.4. Findings of Standard MRI + DWIBS

**Figure 4:**
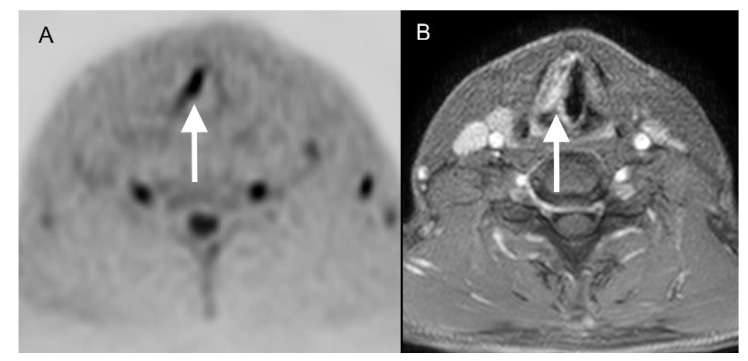
A fifty-year-old patient with a pT1a cN0 cM0 squamous cell carcinoma of the right vocal cord which was detectable for both readers in DWIBS ((**A**); white arrow) as well as in contrast-enhanced transversal T1-weighted scans with fat saturation ((**B**); white arrow).

When considering the setup actually used clinically, comprising standard MRI sequences and DWIBS, Reader 1 identified 30 of 41 vocal cord cancers (73%) correctly and missed the diagnosis in 11 cases (27%). Reader 2 correctly detected 31 cases (76%) and missed 10 vocal cord cancers (24%), respectively (Figure 4). Combined over all readings (n = 82), MRI + DWIBS reduced the number of missed diagnoses from 24 to 21 (−12.5%). The interobserver reliability was calculated as good agreement with κ = 0.69 (*p* < 0.05 (*p* = 0.00001)). The time of acquisition was approximately 11 min, including DWIBS, whereas the standard protocol without DWIBS is approximately 8 min 45 s.

## 4. Discussion

In summary, our own data prove a similarly high detectability rate of glottic laryngeal cancer with standard MRI, DWIBS only, and standard MRI + DWIBS at 68–71%, 63–66%, and 73–76%, respectively. Moreover, interobserver reliability was calculated as good (κ = 0.712), very good (κ = 0.84), and good (κ = 0.69) for standard MRI, DWIBS only, and standard MRI + DWIBS, respectively.

DWIBS improved diagnostic performance by reducing missed cases by 12.5% combined over all readings. DWIBS alone has comparable diagnostic accuracy to standard MRI sequences while showing superior interobserver reliability. 

To the best of our knowledge, no study is testing diagnostic accuracy and interobserver reliability in the diagnostic work-up of patients with early-stage (vocal cord only) laryngeal cancer using DWI or DWIBS imaging techniques. Van Egmond et al. conducted a systematic review of the diagnostic value of MRI for early glottic carcinoma in 2018 and reported concordance between MRI and histopathology in 81% (52 out of 64 patients). In total, 6% (4 out of 64) of tumors were over-staged, and 13% (8 out of 64) were under-staged, but available data for MRI and T1 and T2 glottic carcinomas were very limited as none of the reviewed articles solely included the defined subgroup (cT1 and cT2 glottic carcinomas), and no diagnostic values (sensitivity, specificity, PPV, and NPV) could therefore be calculated [12]. 

Furthermore, the development of dedicated MRI protocols for the evaluation of early-stage laryngeal cancer has not been concluded to date, as scanning protocols vary widely and are not optimal for the diagnosis of early-stage laryngeal cancer. However, implementing diffusion-weighted imaging is highly recommended by most authors [12,23]

MRI is better evaluated for advanced tumor stages, especially for the question of thyroid cartilage infiltration, with sensitivity, specificity, PPV, and NPV varying from 64 to 95%, 56 to 88%, 45 to 73%, and 84 to 96%, respectively [7,24,25,26].

Our homogenous study population of only 41 patients with histopathologically proven vocal-cord-only laryngeal cancer at stage pT1a who had undergone preoperative staging with MRI, including DWIBS, is at the same time both a strong point of the study and its main limitation. Further multicenter studies are required to elaborate on the actual clinical and socio-economic benefits of adding DWIBS to standard MRI protocols. Furthermore, standard DWI sequences are well established and recommended in MRI protocols addressing laryngeal cancer but have not been compared to DWIBS side-by-side in this study. Concerning cost-effectiveness, adding DWIBS to the standard MRI protocol adds 2 min 15 s for a total of 11 min of scanning. As patient preparation and positioning/dismounting account for several additional minutes depending on the setup and patient-individual abilities, adding DWIBS as a standard seems to incur no additional burden on health systems.

## 5. Conclusions

Standard MRI, DWIBS alone, and standard MRI + DWIBS prove an astonishingly satisfactory detection rate as well as convincing interobserver reliability (good, very good, good, respectively) in the diagnosis of early-stage glottic cancer when compared to the definitive histopathologic report.

Larger multicenter and prospective studies should be conducted to confirm our findings and evaluate the true potential of DWIBS, especially compared to standard DWI sequences. 

## Data Availability

Data are available from the authors on request.

## Abbreviations

CT	computed tomography
DWI	diffusion-weighted imaging
DWIBS	diffusion-weighted imaging with background suppression
EPI	echo planar imaging
HPV+	positivity for human papilloma virus
HPV-	negativity for human papilloma virus
MRI	magnetic resonance imaging
MSCT	multislice computed tomography
NPV	negative predictive value
PACS	picture archiving system
PPV	positive predictive value
PET	positron emission tomography
SCC	squamous cell carcinoma
STIR	short T1 inversion recovery
